# Supplementary material for: Avoidable Mortality Attributable to Anthropogenic Fine Particulate Matter (PM2.5) in Australia
Source: Int J Environ Res Public Health. 2020 Dec 31;18(1):254. doi: 10.3390/ijerph18010254 (PMC7795118; doi:10.3390/ijerph18010254)
Supplement: Supplementary file 1 [file ijerph-18-00254-s001.pdf]

All-cause mortality attributable to anthropogenic $PM_{2.5}$ in Australia

Supporting information

S1. Exposure assessment

Concentrations of particulate matter < 2.5 um ($PM_{2.5}$) were estimated from a regression model that used satellite imagery, chemical transport model (CTM) simulation and land use data as predictors, and incorporated direct $PM_{2.5}$ measurements from ambient air monitoring agencies in Australia (1). The data are available on request from Centre for Air pollution, Energy and Health Research. https://cloudstor.aarnet.edu.au/plus/f/2454567279. Annual average $PM_{2.5}$ concentrations were calculated for the centres of Australian Bureau of Statistics (ABS) mesh blocks (MBs) from the 2011 census geography. MBs from 2011 were then assigned to Statistical Area 2's (SA2s) from 2016 to derive population-weighted average exposures.
The maps in Figure S1 show the modelled estimates for 2015 of the annual average $PM_{2.5}$ across the country and inset maps of the greater Sydney metropolitan region and the small case study region in Western Sydney. The Western Sydney inset map shows the MBs and demonstrates the small size of these regions which enables high spatial resolution in our exposure assessment.

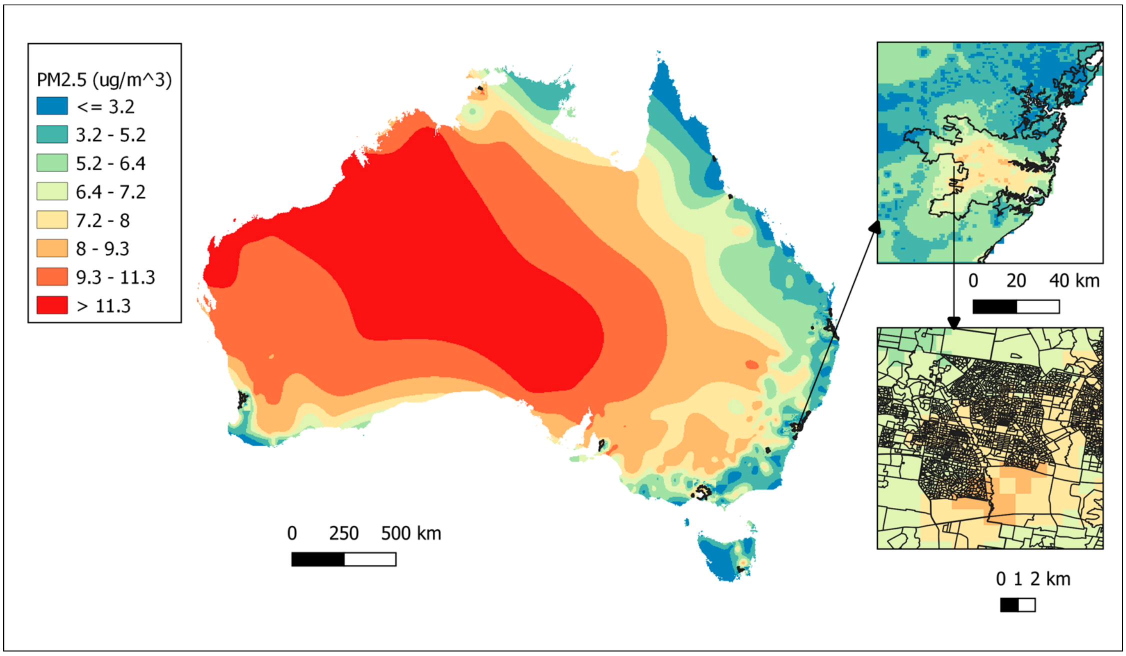

Figure S1. Average $PM_{2.5}$ in 2015 across the country and inset maps of the Sydney region and the small case study region in Western Sydney. The Western Sydney inset map shows the MBs.

S2. Estimated level of anthropogenic $PM_{2.5}$

We estimated the anthropogenic $PM_{2.5}$ as the difference between total $PM_{2.5}$ and the 5th percentile of concentrations observed in any MB per state/territory per

year. Thus, we assumed that the natural background level would be different in each state/territory due to natural processes that may vary with the ecological types found in each state/territory, and the 5th percentile value represents the level there would be without human emissions. Because the Australian Capital Territory (ACT) is a relatively smaller geographic region and the MB range was considered unreliable we used the broader neighbouring region of New South Wales (NSW) to derive the estimate of the non-anthropogenic concentration for that territory.

S3. Health outcomes and population data

We linked each state/territory's age-specific deaths data with the age-specific populations at SA2 by 5-year age ranges (up to age group 85 plus). Mortality data for years 2006-2016 were accessed from the Australian Bureau of Statistics (Cat. No. 3302.0 - Deaths, Australia, http://www.abs.gov.au/AUSSTATS/abs@.nsf/DetailsPage/3302.02016?OpenDocument.

The population data are from Australian Bureau of Statistics (3235.0 - Population by Age and Sex, Regions of Australia - Estimated Residential Population from tablebuilder. Published by the Australian Bureau of Statistics. Retrieved from Centre for Air pollution, energy and health Research https://cloudstor.aarnet.edu.au/plus/f/4179929739). This is the most precise estimate of the population in each year.

S4. Concentration–response functions

The present concentration-response function (CRF) is recommended by the World Health Organization (WHO) Health risks of air pollution in Europe (HRAPIE) project for estimates of all cause long-term mortality due to $PM_{2.5}$ (2).

S5. Life table method

We calculated life tables for each subpopulation as described by Chiang (1968) (3). First the age-specific death rates (M_x) are calculated by dividing the number of deaths in each year by the total number of individuals in each age group. The probability that an individual will die during the age interval (Q_x) is conditional on the fraction of the age interval (0.5) lived by those who die in it and the width of the age interval (5 years). The reciprocal probability of survival (P_x) until the end of the age interval is calculated by subtracting Q_x from 1. These probabilities are then imposed on a hypothetical cohort (I_x) with a starting population of 100000 births and reduced numbers in all age groups according to P_x, and the numbers of deaths in each age group are calculated by subtracting numbers of living people from those of the previous age band. The number of years lived (L_x) in each age interval can then be calculated by adding the number of surviving people in each age group to half the number who died within the 5 year interval. The cumulative number of years lived by the cohort population in the age interval and all subsequent intervals (T_x) is therefore equal to the number of

years lived plus that of the previous age interval. Life expectancy (LE) at the beginning of each age interval is therefore equal to the cumulative number of years lived divided by 100000 (T_x/I_x).

We implemented the calculations using the life tables approach in the IOMLIFETR software by Broome *et al.* (https://github.com/richardbroome2002/iomlifetR) based on the original IOM LIFET system of spreadsheets (https://www.iom-world.org).

S6. LE and years of life lost (YLL)

Using the series of life table matrices to model mortality, we compared LE under a business as usual (BAU) scenario with those of a reduced emissions scenario. Numbers of life years gained by the hypothetical emissions reductions were estimated by calculating the anthropogenic $PM_{2.5}$-attributable numbers of deaths for each age group in the BAU scenario and multiplying these by the remaining LE for each age group and then summing these for all age groups. We report total numbers of life years lost relative to expected life years under the assumption of instantaneous cessation of $PM_{2.5}$. As such, no lag structure has been employed in these calculations. Finally, we averaged LE for children (aged 0–4) in every SA2 across the entire country to indicate the attributable health burden.
YYL across the entire country were calculated for each year of the study using the widely reported equation:

$$YLL = \Sigma(AN_{ij} \times LE_{ij}),$$

where AN_{ij} and LE_{ij} are attributable numbers and the counterfactual life expectancy, respectively, for each age group *i* and SA2 *j*.

S7. Economic calculations

We used the willingness-to-pay value of a statistical life year (VSLY = $213000) as described by the Office of Best Practice Regulation in 2019 (4). VSLY estimates are based on how much people value reductions in the risk of mortality that cumulatively produce an additional life year in a population. We did not adjust this value according to underlying health status, age or LE, but we applied a social discount rate (SDR) of 3% annually. This SDR reflects economic depreciation rather than ageing and is a composite of a private time preference rate, a social time preference rate, the opportunity cost of capital, and a weighted cost of funds discount rate, as described elsewhere (5,6). Whereas VSLY does not vary with age, the value of a statistical life (VSL) does. To calculate age specific VSLs from the VSLY, we applied an annual discount of 3% sequentially for the number of expected remaining life years and summed all discounted values for each age group. As stated in the best practice guidelines (4), the 2019 VSL for a person with 40 years to live is $4.9 million. We calculated age-group specific VSLs by summing sequentially discounted VSLY values for the number of expected remaining life years (LE) of each age group. Age specific VSLs were then multiplied by $PM_{2.5}$-attributable numbers (AN) of deaths.

S8. Sensitivity analysis using climate zones

One of the key assumptions made in our main analysis was the estimate of non-anthropogenic $PM_{2.5}$ concentrations by taking the 5th percentile level within states/territories to represent the lower possible levels that are observed within each geographic region. In alternative analysis for sensitivity assessment we instead used geographic regions that represent climatological boundaries from the Bureau of Meteorology, as shown in Figures S2 and S3. Despite the improved congruity with profiles of seasonal rainfall, which influence periods of increased natural $PM_{2.5}$ from dust and bushfires, our estimates of health burden were similar with 2837 (95%CI 1858, 3746) $PM_{2.5}$-attributable deaths compared to our main analysis estimate of 2616 (95%CI 1712, 3455) using the state/territory based non-anthropogenic $PM_{2.5}$.

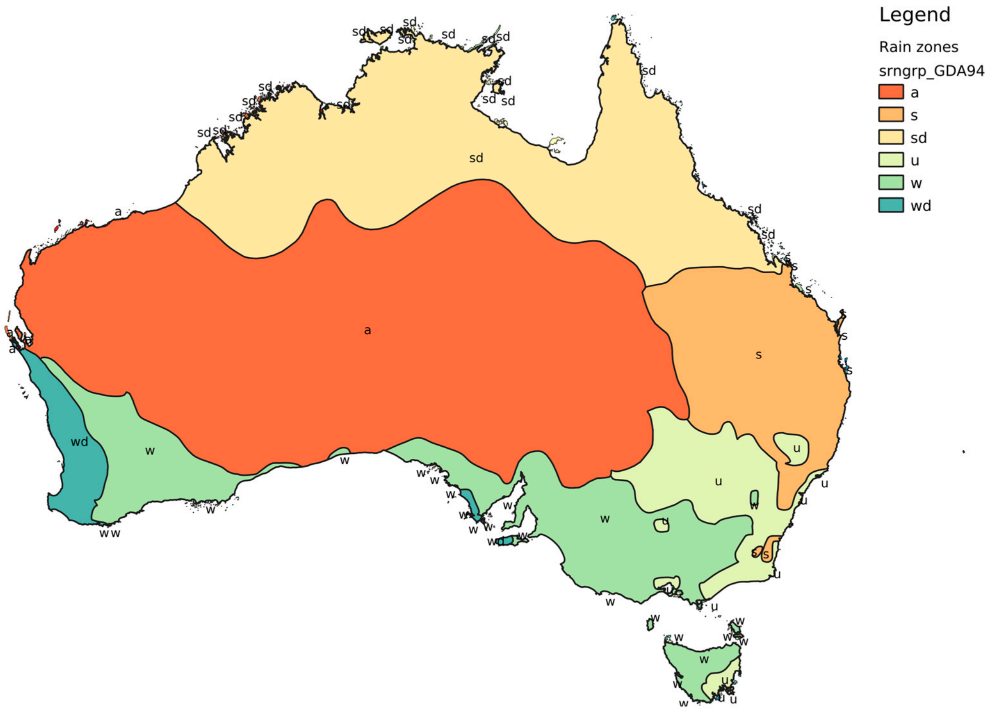

Figure S2: Climate zones from Bureau of Meteorology rainfall levels.
These climate maps are classified by seasonal rainfall and come from the National Climate Centre of the Bureau of Meteorology: Climate Zone Classification. http://www.bom.gov.au/jsp/ncc/climate_averages/climate-classifications/index.jsp. Summer was defined as November - April and winter was defined as May – October. Then the following rules concerning rainfall were followed: Summer dominant (sd), summer > winter AND ratio seasonal rainfall (greater/lesser) > 3 AND Annual total rainfall > 350 mm; Summer (s), summer > winter AND ratio (greater/lesser) < 3 and > 1.3 AND annual total rainfall > 350 mm; Winter dominant (wd), winter > summer AND ratio (greater/lesser) > 3 AND annual total rainfall > 350 mm; Winter (w), winter > summer AND ratio (greater/lesser) < 3 and > 1.3 AND annual total rainfall > 350 mm; Uniform (u), ratio < 1.3 AND annual total rainfall > 350 mm; and Arid (a), annual total rainfall < 350 mm.

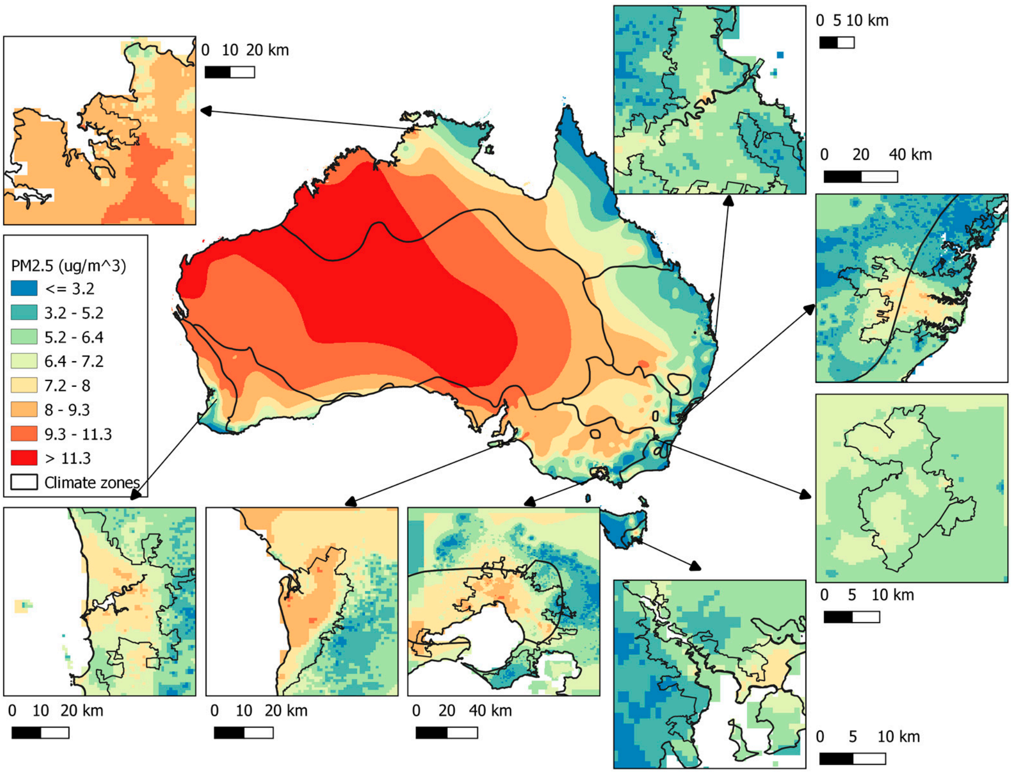

Figure S3: Map of Australia showing modelled estimates of annual average $PM_{2.5}$ (µg/m^3) concentrations in 2015. Climate boundaries are marked by black lines Estimates are based on a spatial smoothing model from all Australian Bureau of Statistics (ABS) mesh blocks (MB) centroids.

S9. Sensitivity analysis using revised relative risk (RR) from recent meta-analysis

A new meta-analysis has recently been published (7) that found support for a RR of 1.08 (95%CI 1.06, 1.09) per 10 µg/m^3 increase in $PM_{2.5}$ which is higher than the RR estimate we have used (2). In an additional sensitivity assessment, we used this new RR and found an increase to our estimated health burden as expected (3335 premature deaths, 95%CI 2534, 3728). However, the difference did not affect our conclusion based on our main analysis that the health burden is substantial.

References.

1. Knibbs LD, Van Donkelaar A, Martin R V., *et al.* Satellite-Based Land-Use Regression for Continental-Scale Long-Term Ambient $PM_{2.5}$ Exposure Assessment in Australia. *Environ Sci Technol*. 2018;52(21):12445-12455. doi:10.1021/acs.est.8b02328

2. WHO. Health risks of air pollution in Europe – HRAPIE project: Recommendations for concentration–response functions for cost–benefit analysis of particulate matter, ozone and nitrogen dioxide. World Health Organization. 2013.

3. Chiang CL. The life table and its construction. In: Introduction to stochastic processes in Biostatistics. New York: John Wiley & Sons, 1968:189–214.

4. Office of Best Practice Regulation. Best Practice Regulation Guidance Note: Value of statistical life. *Heal (San Fr*. 2007).

5. Robinson LA, Hammitt JK, O'Keeffe L. Valuing Mortality Risk Reductions in Global Benefit-Cost Analysis. J Benefit-Cost Anal. 2019. doi:10.1017/bca.2018.26

6. Abelson P. and Dalton T., Choosing the Social Discount Rate for Australia, Aust. Econ. Rev., vol. 51, no. 1, pp. 52–67, 2018.

7. Chen J. and Hoek G., Long-term exposure to PM and all-cause and cause-specific mortality: A systematic review and meta-analysis, Environ. Int., 2020; In Press, doi:/10.1016/j.envint.2020.105974.
